# An automated system for positive reinforcement training of group-housed macaque monkeys at breeding and research facilities

**DOI:** 10.1016/j.jneumeth.2017.04.015

**Published:** 2017-06-15

**Authors:** Jennifer Tulip, Jonas B. Zimmermann, David Farningham, Andrew Jackson

**Affiliations:** a Institute of Neuroscience, Newcastle University, Newcastle NE2 4HH, UK; b MRC Centre for Macaques, Porton Down, Salisbury SP4 0JQ, UK

**Keywords:** Positive reinforcement training, Non-human primate, Automated, Refinement

## Abstract

**Background:**

Behavioural training through positive reinforcement techniques is a well-recognised refinement to laboratory animal welfare. Behavioural neuroscience research requires subjects to be trained to perform repetitions of specific behaviours for food/fluid reward. Some animals fail to perform at a sufficient level, limiting the amount of data that can be collected and increasing the number of animals required for each study.

**New method:**

We have implemented automated positive reinforcement training systems (comprising a button press task with variable levels of difficulty using LED cues and a fluid reward) at the breeding facility and research facility, to compare performance across these different settings, to pre-screen animals for selection and refine training protocols.

**Results:**

Animals learned 1- and 4-choice button tasks within weeks of home enclosure training, with some inter-individual differences. High performance levels (∼200–300 trials per 60 min session at ∼80% correct) were obtained without food or fluid restriction. Moreover, training quickly transferred to a laboratory version of the task. Animals that acquired the task at the breeding facility subsequently performed better both in early home enclosure sessions upon arrival at the research facility, and also in laboratory sessions.

**Comparison with existing method(s):**

Automated systems at the breeding facility may be used to pre-screen animals for suitability for behavioural neuroscience research. In combination with conventional training, both the breeding and research facility systems facilitate acquisition and transference of learning.

**Conclusions:**

Automated systems have the potential to refine training protocols and minimise requirements for food/fluid control.

## Highlights

• An automated positive reinforcement training system for group-housed monkeys.
• Animals can be trained with no food or fluid restriction.
• Breeding facility results may predict subsequent research facility and lab performance.

## 1 Introduction

Positive reinforcement training (PRT) (Skinner, 1938) represents a valuable refinement of laboratory animal husbandry (Prescott and Buchanan-Smith, 2007) and its use with non-human primates (NHPs) is recommended by the UK Home Office, International Primatology Society, National Research Council, Laboratory Animal Science Association and Medical Research Council. PRT can be used to successfully train NHPs to participate voluntarily in procedures (Laule et al., 2003, Young and Cipreste, 2004) such as injection (Priest, 1991), the collection of blood, urine or saliva samples (Priest, 1991, Coleman et al., 2008, Laule et al., 1996, Lambeth et al., 2006, Reinhardt, 2003, Tiefenbacher et al., 2003), movement within/between enclosures (Bloomsmith et al., 1998, Veeder et al., 2009) and may benefit the welfare of captive animals as a result of their ability to control their environment and exercise free choice (Buchanan-Smith and Badihi, 2012). In addition, improved data from co-operative subjects may further reduce the number of animals required for studies (Prescott and Buchanan-Smith, 2007). PRT can provide environmental enrichment for captive animals (Melfi, 2013, Westlund, 2014) and reduce aggression and other behavioural problems in primates (Honess and Martin, 2006, Coleman and Maier, 2010, Minier et al., 2011).

Despite widespread recognition of the benefits of PRT it remains to be universally adopted (Perlman et al., 2012), due to ‘principally a lack of staff and time and a lack of confidence in ability to train’ (Prescott and Buchanan-Smith, 2007). Current PRT regimes are labour intensive, particularly during early stages when it is important that operant behaviours are consistently associated with rewards and performance is systematically documented so that training can proceed at an optimal rate. Consistent daily training has been found most conducive to training success (Fernström et al., 2009).

PRT is commonly used for behavioural neuroscience experiments to train complex cognitive and motor behaviours for food or fluid reward. Animals are typically motivated by restricting corresponding food or fluid intake in the home enclosure (Prescott et al., 2010). While successful in most cases, final performance levels vary considerably across individuals. A small proportion of subjects (∼1 in 10) fail to respond to this routine and must be replaced, incurring welfare costs related to unproductive training, unnecessary transport and re-housing of animals (Davenport et al., 2008, Schapiro et al., 2012).

These considerations motivated us to develop an automated operant conditioning system for unsupervised PRT of NHPs in group home enclosures. While automated systems have previously been reported for use in the training of NHPs (Rumbaugh et al., 1989, Andrews and Rosenblum, 1994, Weed et al., 1999, Mitz et al., 2001, Wilson et al., 2005, Mandell and Sackett, 2008, Fagot and Paleressompoulle, 2009, Truppa et al., 2010, Gazes et al., 2012, Calapai et al., 2016), none of these investigated automated training performance across the different settings in which animals may be trained throughout the course of a typical research project. We investigated the potential benefits of using an automated PRT system at the breeding facility (BF) *before* animals are issued to the research facility (RF) and the effect on subsequent training performance both at the research housing facility and in the laboratory. To do this we designed automated systems which were simple and inexpensive to build/operate/repair, could deliver a high volume of reward and which trained animals to perform a simple motor task using the upper limbs which would be relevant for subsequent research projects. These systems then allowed us to systematically compare animal task performance across the BF, RF and Laboratory settings.

We found automated training enabled animals to learn a high level of performance on simple tasks with minimal requirements for staff time and no food or fluid control, and thus represents a valuable refinement to the training process. In addition, we found that automated training data at the breeding facility correlated with subsequent performance at the research facility and in the laboratory. Thus, automated training records could be used to identify animals suitable for behavioural experiments and assist in optimising the training process for each individual.

## 2 Materials and methods

### 2.1 Automated training systems

**Fig. 1:**
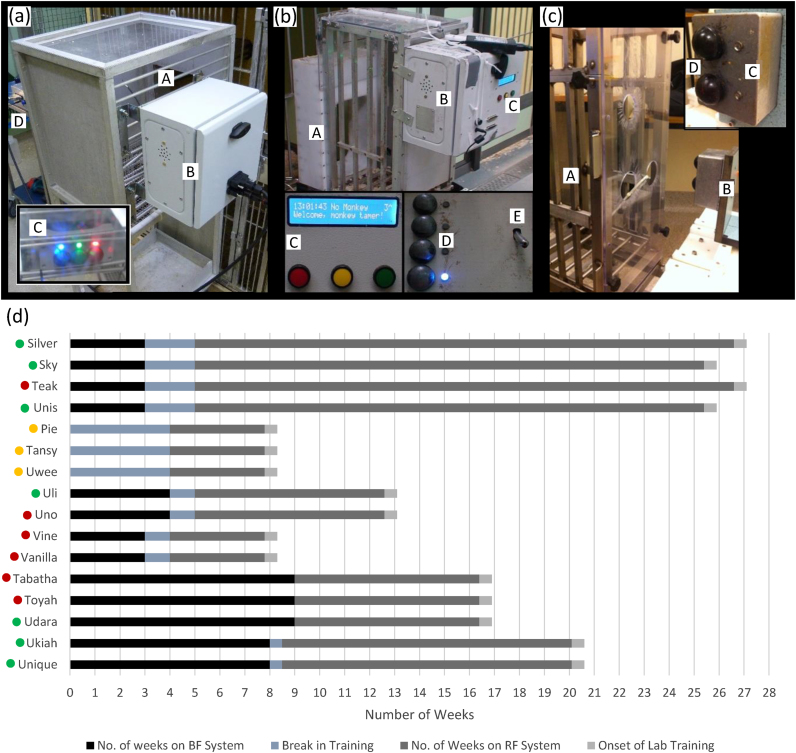
Images of the breeding facility, research facility and Lab training systems. (a) Breeding facility automated training system, comprising (A) transport box, (B) task box, housing (C) coloured cue LEDs and response buttons and also a speaker and a fluid delivery motor pump and spout (not pictured). The control box (D) contains SD card and microcontroller for task control, data collection and storage, and also interaction with technician via a LCD screen. (b) Research facility automated training system, comprising (A) entry tunnel with RFID coil and reader, (B) task box housing SD card, microcontroller, speaker, task electronics, (C) technician user interface, (D) coloured cue LEDs and response buttons, and (E) spout connected to peristaltic pump for fluid reward delivery. (c) Lab training system, for use with a trainer delivering the food-based reward, comprising (A) training chair, (B) task unit consisting of (C) coloured cue LEDs and (D) response buttons. Task unit is connected to a computer for data storage and task parameter control (not pictured). (d) Gannt chart showing the training timelines of each animal across the three training systems until onset of Lab training.

We used three separate systems to collect behavioural data: one at the breeding facility (MRC Centre for Macaques facility at Porton Down, UK), a second in the housing area of the research facility (Newcastle University, UK) and a third for use in the laboratory (Fig. 1).

The systems all comprised coloured LED cues next to response buttons, although the number and physical layout varied slightly across systems (see inset panels in Fig. 1a–c). While some previous systems for automated NHP training have used touch-screens in order to facilitate progression onto tasks of increasing cognitive complexity, (Weed et al., 1999, Mandell and Sackett, 2008, Fagot and Paleressompoulle, 2009, Truppa et al., 2010, Gazes et al., 2012, Calapai et al., 2016), our priority was to design a low-cost (approx. £300 per system), robust system (self-contained and with minimal cables and in-build data storage, easy to clean and repair) that could be used at the BF with minimal support from RF staff located at a different site. Moreover, our animals progress to motor tasks that require interaction with physical devices (levers, manipulanda etc.) rather than cognitive testing. Therefore we designed our system to use robust physical buttons and LED cues, although we do not discount the utility of more advanced technologies (such as touch screens, remote supervision and centralised data storage) for later stages of training on cognitive tasks.

**Fig. 2:**
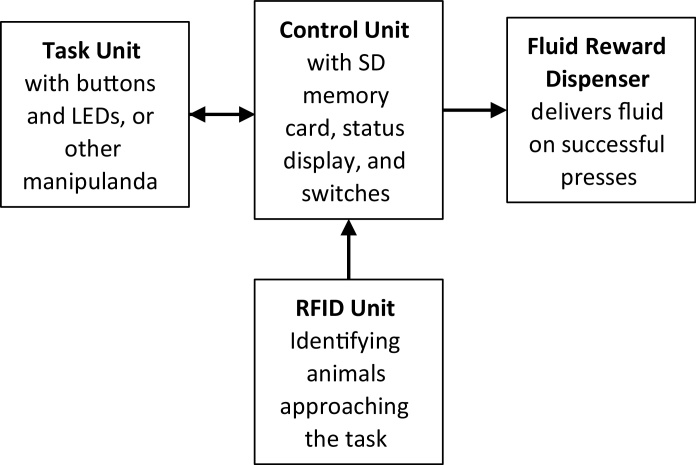
Schematic describing the functional units of the automated systems.

Both BF and RF systems allowed fully-automated training on a button press task cued by coloured LEDs. Fluid rewards (blackcurrant flavour cordial juice, using a 1/10 dilution with water) were delivered via a peristaltic pump and associated with success/error tones delivered via a built-in speaker. A control unit incorporated a microcontroller (ATMega644P, Atmel Corp., San Jose, CA, USA) to run the task, an SD card for data storage, an LCD display and USB serial port for exporting data to a PC (Fig. 2).

Whenever a LED cue was illuminated, animals received either a “success” tone and fluid reward for pressing the corresponding button continuously for a defined hold time, or an “error” tone and no fluid reward for pressing an incorrect button or releasing the correct button too soon. Following any incorrect press was an adjustable time out period where no LEDs were illuminated and no reward dispensed. The time out period was prolonged if any button presses were attempted during this time, thus enforcing the importance of the LED cues for successful reward retrieval and discouraging random button presses. Following an incorrect attempt, the same LED cue would illuminate again. This same LED would continue to illuminate until correctly pressed, to discourage stereotyped pressing of only one button. Correct and incorrect responses were recorded to an SD memory card along with the date, time and task parameters. The breeding facility system fitted directly onto an annex cage situated off the main NHP group enclosure (Fig. 1a). The research facility system was attached to a standard training chair situated off the animals’ enclosure (Fig. 1b). The same training chair was subsequently used to bring animals to the laboratory (Fig. 1c). Unlike the fully-automated systems, training in the laboratory used food reward (assorted chopped fruit, dried fruit and nuts) hand-delivered by the trainer. LED cues were controlled by a PC which also provided success/error tones and recorded performance data.

The research facility system also incorporated a sub-cutaneous radio-frequency identification (RFID) reader coil to automatically identify animals as they entered the training chair. Sessions were filmed at least twice per week and we compared visual identification of animals against the electronic records to monitor the reliability of the RFID reader recognition rate, which in the short term performed at >80%. However, due to hardware issues RFID identification was only used for four animals; the remainder were individually separated for training sessions.

### 2.2 Dataset

We collected data from 16 female rhesus macaques (Table 1) that required behavioural training as part of several ongoing neuroscience projects at the research facility. All regulated procedures were approved by local ethics committee and performed under appropriate UK Home Office licenses in accordance with the Animals (Scientific Procedures) Act 1986.

**Table 1:** Details of the animals involved in the project. Housing groups remained the same throughout the time period described in the results section with the exception of group 1, who were divided into two pairs (indicated by dashed line). Ages and weights given here are those recorded at onset of research facility training for each individual. An individual’s position within the social hierarchy is described as Dominant (Dom), Mid-Rank (Mid) or Subordinate (Sub). For the largest group, group 1, the mid-rank animals are further classified as upper or lower mid-rank. Column 6 indicates paternal half-sister relationships. Columns 7–9 detail the exposure to and performance on breeding facility (BF) system.

Housing Group	Name of Animals in Group	Age	Weight (Kg)	Position in social hierarchy	Related to cage-mates?	Exposed to BF system and did use it	Exposed to BF system and did not use it	Unexposed to BF system
1	Silver	3y 11m	5.40	Dom	Teak	✓		
	Teak	3y 4m	5.13	Mid (lower)	Silver	✓		
	Sky	3y 10m	5.82	Mid (upper)	Unis	✓		
	Unis	2y 4m	3.35	Sub	Sky	✓		
2	Pie	6y 8m	7.70	Dom	Uwee			✓
	Tansy	3y 5m	5.31	Mid	–			✓
	Uwee	2y 6m	4.75	Sub	Pie			✓
3	Uli	3y 2m	4.70	Dom	–	✓		
	Uno	3y 1m	4.50	Sub	–		✓	
4	Vine	2y 2m	3.37	Sub	–		✓	
	Vanilla	2y 2m	3.69	Dom	–		✓	
5	Tabatha	5y 2m	6.24	Sub	–		✓	
	Toyah	5y 2m	7.56	Dom	Udara		✓	
	Udara	4y 0m	6.58	Mid	Toyah	✓		
6	Ukiah	4y 2m	6.98	Sub	–	✓		
	Unique	4y 2m	6.85	Dom	–	✓		

Animals were group-housed at the breeding facility (8–13 animals per group) and the research facility (2–4 animals per group). During the training period reported here, all animals had *ad libitum* access to water and standard diet consisting of three types of pellets (Old World Monkey Standard/Chunks/Trio Munch, Special Diets Services, Witham, Essex, UK) and forage mix (Lillico Forage Mix, Lillico Biotechnology, Hookwood, Surrey, UK). At the breeding facility, the animals are fed twice daily, where pellets were given in the morning (between 8:30-10am) and forage mix was given in the afternoon (between 12-2pm). In exception to this routine, any “juicy” foods such as fruit were given after training had been completed each day. At the research facility, pellets and forage were refreshed each morning and afternoon, and assorted fruit was given each Friday afternoon after training had been completed.

Due to varying requirements of the different neurophysiology research projects, the animals in this study received varying amounts of behavioural training. However, all animals received a minimum of at least:

• 1 week of group exposure and 2 weeks of individual exposure to breeding facility automated system (or alternatively no exposure at all at breeding facility)
• 27 sessions using the research facility automated system
• 8 sessions using the lab system

### 2.3 Training protocols

**Fig. 3:**
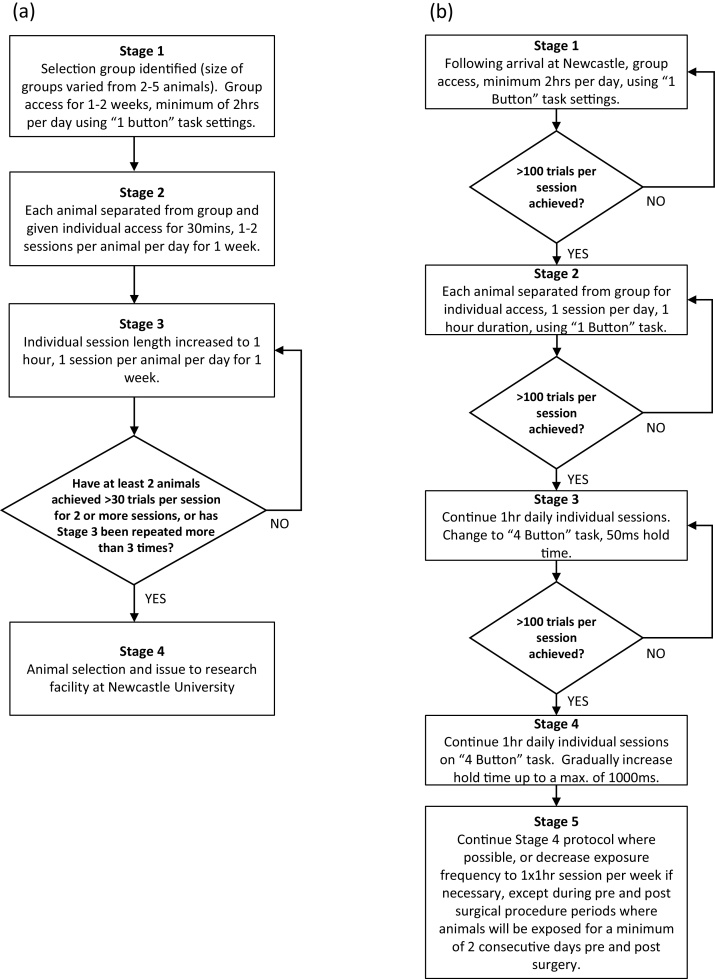
Exposure and training protocol flow charts. Charts Illustrate how animals progress through their training on (a) breeding facility system and (b) the Newcastle research facility system.

Fig. 3a shows the breeding facility training protocol. Initially, preselected groups of 2–5 animals were allowed unsupervised access to the system for 2 h daily (excluding weekends), using a “1 button task” which rewarded a press of any button. In some cases, buttons were ‘baited’ with food to encourage interaction with the system. This introductory period lasted until at least some animals were pressing buttons (typically 1 week). Subsequently individuals were separated to record their individual performance during daily 30–60 min sessions for a minimum of 2 weeks. After this, animals were transported to the research facility (irrespective of their performance).

Fig. 3b shows the research facility training protocol and criteria for progression from “1 button task” to a “4 button task” in which animals were rewarded only for pressing a single cued button. Animals were given access to the system from their second week at the facility. Animals received daily training sessions (excluding weekends). The initial task settings were those the animal last used at the breeding facility before being issued to Newcastle.

Initially laboratory training was restricted to short 10 min sessions which were gradually increased over 8 days to a maximum of 36 min per session (with some variability due to the animals’ response to laboratory training). The first session in the lab began using the “1 button task” after which the animals progressed to a “2 button task”. To avoid unconscious bias, the training staff were blinded to breeding facility data until after the animals had completed the entire research facility and laboratory training protocol.

### 2.4 Breeding facility performance classification

**Fig. 4:**
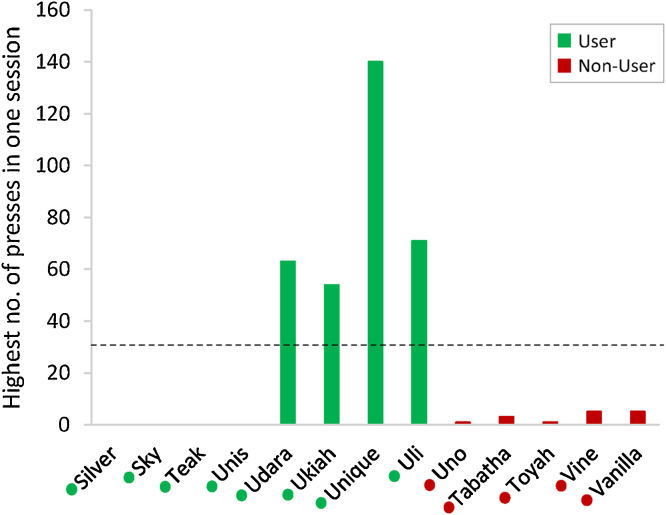
Highest performance scores of each individual in any single session on the breeding facility system. To be classified as a “User”, an individual had to perform at least 30 successful presses (dashed line) in at least 1 session. Silver, Sky, Teak and Unis data was lost, but it is known that they performed at least 50 successful presses. As we can see here, Silver – Uli would be classed as “Users” while Uno – Vanilla would be classed as “Non-Users”. Groupings indicated by coloured dots next to names (green = User, red = Non-User) (For interpretation of the references to colour in this figure legend, the reader is referred to the web version of this article.).

Animals were classified as “Users”, “Non-Users” or “Unexposed” based on performance at the breeding facility (Fig. 4). “Users” performed ≥30 successful button presses in at least one session (this criterion was chosen to distinguish genuine system use from occasional accidental presses), while animals that failed to reach this criterion were classified as “Non-Users”. One group of animals received no training at the breeding facility were classified as “Unexposed”. Due to hardware issues, the breeding facility performance data for the first four animals (Silver, Sky, Teak and Unis) was corrupted, but communication at the time with breeding facility staff confirmed that all four animals satisfied performance requirements and were included in the analysis as Users.

### 2.5 Research facility and laboratory performance measures

Performance within a single session was quantified using two measures: the rate of successful presses per hour, and the percentage of all presses that were successful and therefore received reward. Note that for an attempt to be rewarded, the button had to be pressed continuously for 50 ms. Therefore, unsuccessful attempts include both presses of the wrong button and presses of the correct button for an insufficient duration. As a result, the percentage successful presses can be less than 100% even for the “1 button task”.

### 2.6 Time windows for behavioural analysis

We compared the research facility performance of User, Non-User and Unexposed groups (classified by breeding facility performance) over two time periods, aligned to different events in the training protocol. The first time period began with the 5th training session after arrival at the research facility (the first 4 sessions were discarded since individual data was not available for all animals) and ended with the 27th training session (the last session for which we had data for all 16 animals). The second time period began with the first session at which animals were moved to the “4 button task”, and ended with the 13th session on this task (the maximum that allowed us to incorporate data from all animals). To assess performance with the laboratory system, we used a single time window consisting of the first 8 sessions of training in the lab. In each case we calculated average performance within groups in each session, and also the average performance across all sessions within the time period.

Analyses were performed in Matlab, Microsoft Excel and Minitab 17. Statistical tests assumed each animal as an independent observation and we performed non-parametric analyses to avoid assumptions of normality.

## 3 Results

### 3.1 Automated PRT can train simple tasks with no food or fluid control

**Fig. 5:**
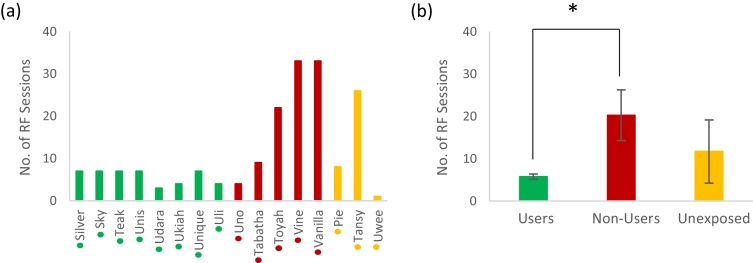
Comparing the number of research facility (RF) system sessions required in order to attain a specified level of performance, using comparison groups based on previous breeding facility performance. Individuals classified as either Users (green), Non-Users (red) or Unexposed (orange) based on breeding facility performance. (a) shows the number of sessions of each individual to meet the performance criterion “≥100 correct presses on research facility system regardless of task setting” (dots below names on *x* axis indicate group classification). (b) shows the overall group mean number of sessions required to fulfil that same performance criterion for each of the three groups (error bars show standard error, * indicates significance P < 0.05) (For interpretation of the references to colour in this figure legend, the reader is referred to the web version of this article.).

In general, animals quickly began to interact with the automated feeding system and started pressing buttons, despite being under no food or fluid control. Fig. 5a shows the number of training sessions at the research facility needed by each animal before they successfully performed ≥100 correct presses per session. Note that all animals achieved this level of performance, although the requisite amount of training varied from 1 to 33 sessions.

### 3.2 Breeding facility performance predicts research facility performance on simple tasks

Fig. 5b shows the average number of training sessions required by Users, Non-users and Unexposed groups classified according to breeding facility performance. The Users required a mean (± standard error) of only 6 ± 1 sessions, compared with 12 ± 8 sessions for the Unexposed group and 20 ± 6 sessions for Non-users. Although a Kruskal-Wallis test did not find the difference between groups to be significant (H_2_ = 4.66, P = 0.097), a Mann-Whitney test (U = 6, P = 0.048) confirmed a significant difference between the means of the Users and Non-Users groups.

**Fig. 6:**
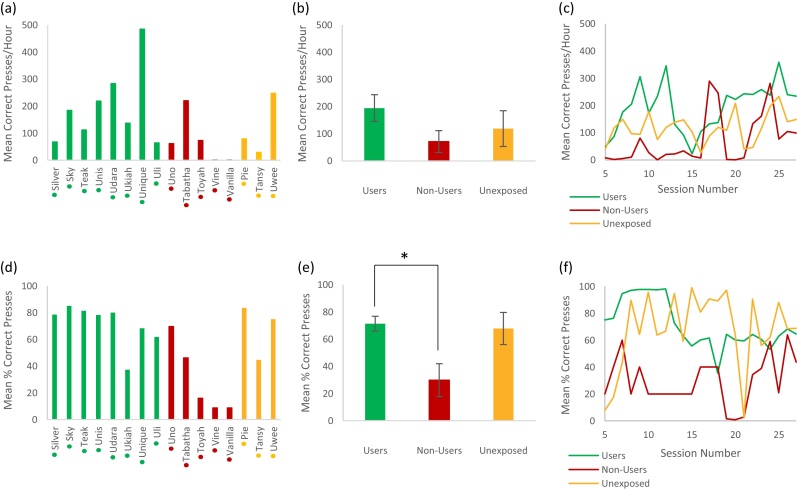
Performance on the research facility system over sessions 5–27 of exposure, using categorisation into groups based on previous breeding facility performance. Individuals sorted into one of three groups depending on their breeding facility performance – Users (green), Non-Users (red) and Unexposed (orange). (a), (b) and (c) use number of correct presses per hour as the research facility performance measure, while (d), (e) and (f) use% correct presses as the research facility performance measure. (a) and (d) show the mean performance of each individual across all sessions. Coloured dots next to x axis names indicate User/Non-User/Unexposed group classification. (b) and (e) show the overall group means across all sessions (error bars show standard error and * indicates P < 0.05). (c) and (f) show the learning curves of each group over sessions 5–27 on the research facility task based on the mean group performance for each session (For interpretation of the references to colour in this figure legend, the reader is referred to the web version of this article.).

Next we asked whether an animal’s breeding facility classification (User/Non-User/Unexposed) influenced performance on the research facility system over a time-window covering the 5th to 27th training sessions (Fig. 6). Fig. 6a shows the average successful presses per hour for each individual across all sessions. Fig. 6b shows average performance for each classification group across all sessions and Fig. 6c shows the group average learning curves over the sessions. The rate of successful button presses increases throughout the training period, with Users having the highest mean (±standard error) rate of 194 ± 49/h, followed by the Unexposed group (119 ± 66/h) and then Non-Users (71 ± 40/h). A Kruskal-Wallis test (H_2_ = 3.54, P = 0.17) fails to show a significant difference between groups. However, a Mann-Whitney test (U = 8, P = 0.09) implies a weak trend to suggest Users perform at a higher rate than Non-Users.

Overall these results suggest that animals who are classified as Users on the breeding facility system go on to perform to a higher standard at Newcastle on the research facility system and achieve a competent performance level sooner than those classified as Non-Users. This suggests that training at the breeding facility can transfer to the research facility.

### 3.3 Breeding facility performance does not predict progression to more complex tasks

**Fig. 7:**
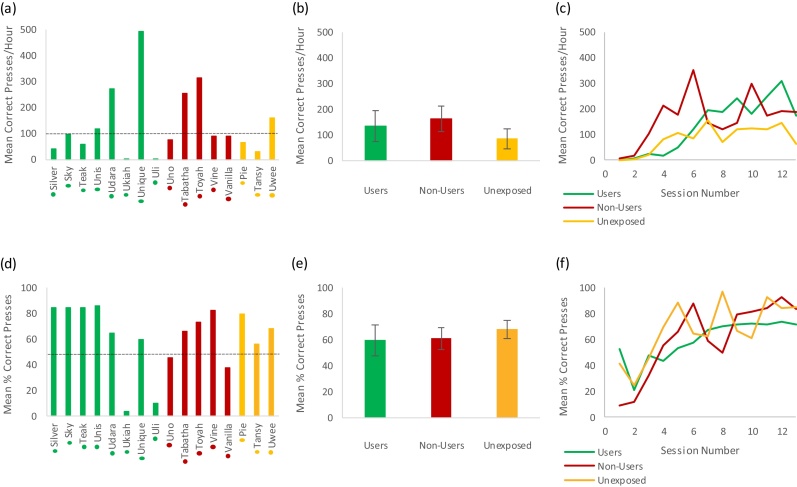
Performance on the research facility system over the first 13 sessions using the 4 button task setting, using categorisation into groups based on previous breeding facility performance. Individuals sorted into one of three groups depending on their breeding facility performance – Users (green), Non-Users (red) and Unexposed (orange). (a), (b) and (c) use number of correct presses per hour as the research facility performance measure, while (d), (e) and (f) use% correct presses as the research facility performance measure. (a) and (d) show the mean performance of each individual across all sessions. Coloured dots next to x axis names indicate User/Non-User/Unexposed group classification. (b) and (e) show the overall group means across all sessions (error bars show standard error). (c) and (f) show the learning curves of each group over 13 sessions on the research facility 4 button task based on the mean group performance for each session (For interpretation of the references to colour in this figure legend, the reader is referred to the web version of this article.).

Since the research facility training protocols was designed to progress animals on to the more challenging 4-button task at different time points only once they had acquired the 1-button task (defined as >100 successful presses per hour; Fig. 5a), we performed a separate analysis of performance subsequent to this event (Fig. 7). For this measure there was no clear difference between the progress of each group (Fig. 7a–c). The average rate of successful presses was 135 ± 60/h, 164 ± 50/h and 85 ± 39 h for Users, Non-users and Unexposed groups respectively (Kruskal-Wallis test: H_2_ = 1.23, P = 0.54). A Mann-Whitney test (U = 15, P = 0.51) confirmed a lack of significant difference between the Users and Non-Users groups. In general, most animals quickly began performing above chance levels at the 4-button task. The percentage of attempted presses that were successful (Fig. 7d–f) was similar between groups (Users: 60 ± 12%, Non-Users: 61 ± 8%, Unexposed: 68 ± 7%, Kruskal-Wallis test: H_2_ = 0.47, P = 0.79; Mann-Whitney test Users vs. Non-users: U = 16, P = 0.61). In summary, these results suggests that breeding facility performance does not predict the rate at which animals improve during later training stages when they move on to the 4 button task. It is important to note however that the time required to get the animals to this stage of training was longer for the Non-users compared to Users. In addition, progression from the 1-button to 4-button tasks generally required only a few sessions (∼1 week) before animals were performing at a high level and this was again achieved without food or fluid control.

### 3.4 Breeding facility performance and research facility training predict performance on the laboratory task

**Fig. 8:**
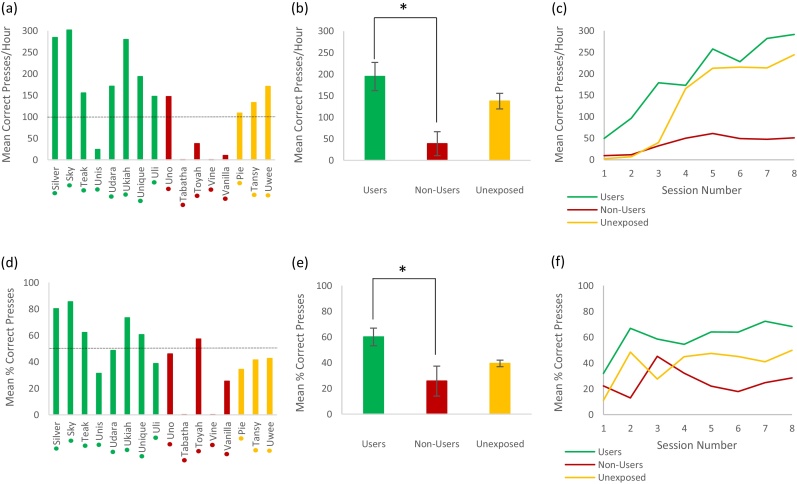
Performance on the Lab system over the first 8 sessions of exposure to the system, using categorisation into groups based on previous breeding facility performance. Individuals sorted into one of three groups depending on their breeding facility performance – Users (green), Non-Users (red) and Unexposed (orange). (a), (b) and (c) use number of correct presses per hour as the Lab performance measure, while (d), (e) and (f) use% correct presses as the Lab performance measure. (a) and (d) show the mean performance of each individual across all 8 sessions. Coloured dots next to x axis names indicate User/Non-User/Unexposed group classification. (b) and (e) show the overall group means across all 8 sessions (error bars show standard error and * indicates P < 0.05). (c) and (f) show the learning curves of each group over 8 sessions on the Lab task based on the mean group performance for each session (For interpretation of the references to colour in this figure legend, the reader is referred to the web version of this article.).

Next we assessed whether breeding facility classification influenced the performance of animals on a similar button-press task in the laboratory. The performance data for the first 8 sessions using the lab system is illustrated in Fig. 8. There was a significant influence of breeding facility classification on the mean rate of successful presses (Users: 195 ± 33 h, Non-users: 39 ± 28/h, Unexposed: 138 ± 18/h; Kruskal-Wallis: H_2_ = 8.36, P = 0.015; Mann-Whitney test Users vs. Non-users: U = 2, P = 0.01). Similar results were obtained for the percentage of attempted presses that were successful (Users: 60 ± 19%, Non-users: 26 ± 26%, Unexposed: 39 ± 4%; Kruskal-Wallis: H_2_ = 5.52, P = 0.06; Mann-Whitney test Users vs. Non-users: U = 5, P = 0.03).

The results of the preceding section suggest that breeding facility performance influenced subsequent lab performance, with Users out-performing Non-users over the early training stages of the laboratory-based task. However, unlike the training protocol using the automated system which was identical for all animals, the timing of laboratory training was influenced by other factors. For example, animals were only taken to the lab environment after they had successfully completed “chair training” (which used positive reinforcement to train animals to move voluntarily between enclosure sections and into the smaller training chairs). Moreover the beginning of laboratory training was influenced by the needs of ongoing neuroscience projects to which the animals were pre-allocated. Therefore the onset of lab training could not be controlled and we examined whether this variable also influenced subsequent Lab performance.

**Fig. 9:**
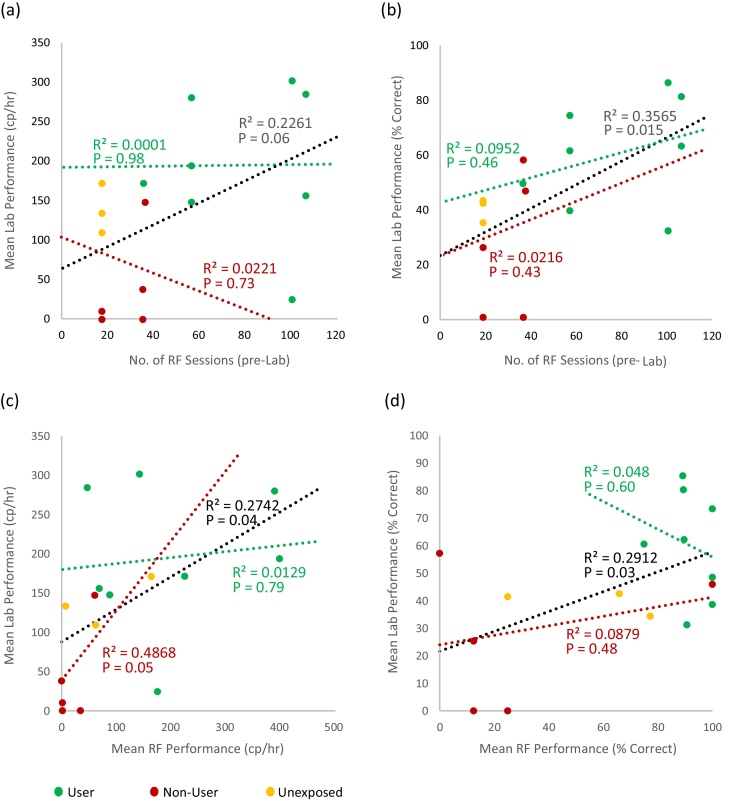
Correlation between number of pre-Lab research facility (RF) system sessions (i.e. amount of practice) and subsequent Lab system performance. In all plots, each data point is colour-coded to indicate whether the individual had practice on the task at the BF (a User; green), or had not used the BF system and therefore had no practice on the task (Non-users and Unexposed; red and yellow). Linear regression plots compare number of pre-Lab research facility sessions to mean Lab performance over the first 8 Lab sessions for each individual, using (a) correct presses per hour and (b) % correct presses as performance measures. Panels (c) and (d) show linear regression plots comparing the performance during early RF sessions (sessions 5–12) and Lab performance (first 8 sessions), using (c) correct presses per hour and (d) % correct presses as performance measures. In all plots, linear regression analysis was carried out on the dataset as a whole (black line), and on the User (green line) and Non-user/Unexposed (red line) groups individually.

Fig. 9a,b shows the total number of research facility system training sessions preceding the onset of Lab training compared against laboratory performance measured as the mean rate of successful presses and the mean percentage of attempted presses that were successful. Linear regression analysis across all subjects (Fig. 9 black lines) suggested a possible influence of amount of practice on both measures (rate of successful presses: R^2^ = 0.23, P = 0.06; percentage successful: R^2^ = 0.36, P = 0.015). However, the User animals also tended to receive higher numbers of pre-Lab sessions. This makes it is difficult to distinguish whether the apparent trend in the data is due to amount of pre-lab practice at the RF, different BF experiences, or both of these factors. We therefore divided the group into those animals who had performed the task at the BF and therefore gained BF experience (the Users), and those who had not performed at the BF (either by being Unexposed or being a Non-user). We performed linear regression analysis for these two groups separately to investigate whether the within-group variability in laboratory performance could be explained by RF practice (i.e. no. of pre-Lab sessions). If BF experience made no difference to subsequent RF performance, we would expect both groups to fall on the same trend-line, whereas if BF experience alone determined subsequent laboratory performance we would expect both groups to be fit by horizontal lines with different intercepts. The red/green regression fits in Fig. 9a,b indicate no significant correlation within groups between number of RF sessions and laboratory performance measured by “cp/hr” (R^2^ = 0.0001, P = 0.98; Non-users/Unexposed: R^2^ = 0.02, P = 0.73) or “% Correct” (Users: R^2^ = 0.095, P = 0.46; Non-users/Unexposed: R^2^ = 0.02, P = 0.43). Moreover, the linear fit for Users is higher than Non-users/Unexposed irrespective of the number of RF sessions. However, reduced sample size means there is a reduced statistical power for these within group correlations, and the data for “% Correct” at least appears consistent with an effect of amount of RF training in both cases.

As a final analysis we examined whether, irrespective of the total number of RF training sessions, performance in early RF sessions was predictive of performance in later laboratory training (Fig. 9c,d). We chose 8 sessions early in RF exposure (sessions 5–12) to compare against the 8 laboratory training sessions, in effect comparing performance during equivalent periods of task performance in a novel context. We observed robust correlation across the entire dataset for both the “cp/hr” (R^2^ = 0.27, P = 0.04) and “% Correct” (R^2^ = 0.29, P = 0.03). Moreover, amongst animals that had not performed the task at the BF (Unexposed and Non-users), there was a significant correlation for the “cp/hr” group (R^2^ = 0.49, P = 0.05). Note that this group of animals were similar in terms of both their lack of task practice at the BF and the number of pre-lab RF training sessions they received. Therefore, this correlation supports the conclusion that the early pre-lab RF training data can be used to predict subsequent laboratory performance.

In summary, our data demonstrates that use of the automated PRT system can facilitate subsequent laboratory training, and that automated records obtained at both the BF and RF can be used to predict laboratory performance. However, a larger dataset with more control over the timing of laboratory training will be needed to distinguish whether individual characteristics (i.e. User vs. Non-user temperament), or the amount of pre-training afforded by the automated system at either the BF or RF has the greatest influence on subsequent performance levels.

## 4 Discussion

### 4.1 An automated system for PRT with group-housed monkeys

We have described a simple and robust automated system for positive reinforcement training of group-housed non-human primates that was used at both a breeding facility and research facility. We have shown that automated PRT can be used to train a cohort of animals to perform a simple cued button press task at consistent level. By making the system accessible from the home-enclosure of group-housed macaques, their natural curiosity is sufficient to motivate learning without the need for food or fluid control. Moreover, task skill acquired at the breeding facility transfers to facilitate early stages of training at the research facility and in the laboratory, and recorded data at each stage can be used to predict early performance in each new context. We will first discuss the benefits of employing such training, before considering some of the limitations of our approach.

### 4.2 Benefits of automated PRT at the breeding facility

To our knowledge, this is the first study to initiate automated PRT training at a breeding centre *before* animals are issued to a neuroscience facility. We observed variability in the extent to which individuals interacted with the system during this early training, and this correlated with subsequent performance metrics at the research facility and in the laboratory. From our study, it is not possible to determine whether the differences between Users and Non-users reflect intrinsic characteristics of animals such as temperament and cognitive ability, or their social relationships within the group. It is possible that the time that animals start using the system is due to random chance, and as a result Users simply accumulate more experience at the BF and then perform better at the RF and in the lab. However, Unexposed animals typically performed somewhere between Users and Non-Users on most RF and laboratory metrics (albeit this was a small sample size) and in early sessions at the RF facility, the best performing Unexposed animal (Uwee in Fig. 5, Fig. 6 was already reaching a level comparable to most Users. Moreover, across Non-Users and Unexposed animals, the percentage of correct button presses in early RF sessions and laboratory training were correlated, despite all these animals lacking practice at the BF. This suggests prior practice at the BF may not alone explain the gap between Users and Non-Users upon reaching the research facility.

Once animals reached the requisite performance level to move onto the 4-button version of the task, animals that were Users, Non-users and Unexposed at the breeding facility system progressed at comparable rates within the now familiar RF home-cage environment, suggesting that our classification may not reflect general cognitive ability. However Coleman et al. (2005) noted that animals reluctant to approach a novel object subsequently trained slower than the more confident individuals. Therefore breeding facility classification may indicate confidence or curiosity in relation to novelty (Chamove, 1983, Capitanio et al., 1986, Boccia et al., 1995). Such an interpretation would explain the observed relationships between breeding facility classification (when first exposed to the device), early performance at the RF (when encountering the device in a new environment) and the early stages of laboratory training (entailing a novel environment and interaction with a human trainer), all of which could act as potential stressors (Morgan and Tromborg, 2007). However, further studies would be required to examine directly whether response to novelty explains our BF classification and is related to the genetic profile or social ranking of individual animals.

Whatever the underlying cause of different BF classifications, the fact that Users of the system subsequently performed at a higher standard than Non-users during early training at the research facility and in the laboratory suggests that automated performance data is useful for gauging how quickly animals will transition through a training programme. Behavioural training regimes for many neuroscience experiments entail the introduction of further novel elements throughout, including more complex tasks, restraint and experimental techniques required to record from the brain. Moreover, training time is a major constraint in NHP neuroscience experiments, in terms of the time required to perform studies, the staff resources required and the welfare costs associated with time spent by animals on fluid/food control protocols. The differences we observed in this study at an early stage suggest automated PRT at the breeding facility could serve as an indicator of how much time will be required to train each individual subsequently, allowing animals to be matched with appropriate studies (for example, allocating animals defined as Users onto projects requiring rapid behavioural progression and allocating Non-users onto projects where behavioural training is unnecessary or can proceed at a slower pace). However, if characteristics determining BF classification are hereditary (Champoux et al., 2002), it will be important to ensure that any selection of animals for behavioural experiments does not restrict the heterogeneity of animals used for breeding subsequent generations within colonies. Even if not used for selection of animals, beginning automated PRT at the breeding facility may be beneficial in providing a familiar environmental feature once animals are moved to the research facility, enabling them to exercise control over the new environment (Buchanan-Smith and Badihi, 2012). Moreover, since voluntary use of the system was achieved without food or fluid control, animals are likely motivated by curiosity rather than dietary need. Impoverished environments can have a negative impact on welfare (Morgan and Tromborg, 2007, Young, 2003), and the process of training can provide environmental enrichment (Melfi, 2013, Westlund, 2014). Finally, the associations trained by automated PRT may subsequently be shaped into other behaviours that may be useful within a breeding facility, such as moving between enclosures, stationing for examination/injections etc.

### 4.3 Benefits of automated PRT at the research facility

The early stages of behavioural training necessary for neuroscience research projects is labour intensive and involves a number of potential stressors including adaptation to a new laboratory environment and human interaction (Morgan and Tromborg, 2007). As a result such training is often motivated by food or fluid control. Automated PRT within the home-enclosure at the research facility can facilitate this process by allowing animals to become accustomed to the transport chair and basic task requirements with a minimum of human involvement and no food/fluid control. Task parameters can be altered to increase task difficulty and enable progression of training from a 1-button to 4-button task at the appropriate stage for each animal. Moreover, home-enclosure performance can be used as an indicator of when an individual is ready to begin laboratory training. In our experience, better time management of training regimes reduces the need for non-PRT techniques to keep animals on schedule, and reduces likelihood of disruptive behaviours during training (such as refusing to cooperate or struggling against restraints). As a result, once animals enter the laboratory, they are able to progress more quickly through early stages without the frustration and stress of unfamiliar task requirements. We were therefore able to achieve several hundred successful button presses per hour in the laboratory after 8 sessions of training. While additional motivation may be necessary for more complex tasks, our results show that an appropriate training regime incorporating automated home-enclosure PRT is capable of implementing the early stages of laboratory training without food or fluid control and therefore reflects a welfare refinement with minimal cost in terms of staff time or resources.

### 4.4 Limitations of automated PRT

While we believe that automated PRT can be a valuable component of behavioural training regimes, there are important limitations to what can be achieved with our system. In initial exposure sessions, group-housed animals were free to enter and leave the training cage at will, which may be advantageous since one curious animal may be followed by cage-mates who might otherwise be wary of entering the training cage. However, while the RFID reader system was capable of identifying animals as they entered, we were unable to attribute button presses to specific animals if more than one entered together. As a result of this (and reliability issues with the RFID reader), individual training sessions (where one animal was separated from the group) were used to collect most of the data presented here. This issue could be addressed by redesigning the interface to facilitate identification of the particular animal pressing buttons and/or redesigning the entrance to allow only one animal through at a time. However, such modifications come at the cost of increased system complexity. A second limitation is that an automated system can lead to uncontrolled variation in button pressing technique. In extreme cases, some animals in this study were observed to press the buttons with their mouth to obtain reward. Again, a different design of interface could constrain the rewarded behaviour, e.g. by recessing the buttons into a channel that is too narrow for the animal’s head. Adding a tool to allow for remote supervision could also address this issue, since it is inevitable that some monitoring by a human trainer is advisable to ensure that incorrect techniques are not repeatedly reinforced, resulting in training of unwanted behaviours that are of no use in the laboratory. Third, the current design of our system is limited to a simple button press, cued by LEDs. This was motivated by the desire to develop a robust system, self-contained unit suitable for use at a remote breeding facility. Our research interests are in motor control, and therefore our animals progressed straight from this task to upper-limb movement tasks. Nevertheless, it would be relatively simple to transition to a more complex visual display for cognitive tasks if required. Finally, it is worth noting that interaction with human trainers and husbandry staff is an important aspect of adaptation to laboratory routines and we do not suggest that automated systems can or should replace such interaction completely. However, we believe such interaction can be facilitated if human training exploits the associations that have already been developed using the automated system.

## 5 Conclusions

We have implemented automated positive reinforcement training at a breeding facility (CFM) and research facility (Newcastle University CBC). 16 female rhesus macaques attained a high level of performance with minimal staff time and without any need for food or fluid control. Moreover, automated training data at the breeding facility correlated with subsequent performance at the research facility and in the laboratory, suggesting that automated training records could be used to identify animals suitable for behavioural experiments and assist in optimising the training process for each individual. Potential future investigations of the role of social learning and animal temperament could facilitate refinement of training protocols which are better tailored to individual animal needs and indicate when they are ready to progress to the next stage of training. Finally, aside from the training benefits of automated PRT, sustained voluntary use of the systems in the absence of any dietary need suggests a potential application as environmental enrichment.
